# Supplementary material for: Genome-Wide Identification, Expansion, and Evolution Analysis of Homeobox Gene Family Reveals TALE Genes Important for Secondary Cell Wall Biosynthesis in Moso Bamboo (Phyllostachys edulis)
Source: Int J Mol Sci. 2022 Apr 8;23(8):4112. doi: 10.3390/ijms23084112 (PMC9032839; doi:10.3390/ijms23084112)
Supplement: Supplementary file 1 [file ijms-23-04112-s001.zip › Table S2.pdf]

Table S2 Gene symbol of TALE genes in moso bamboo.

Gene_sym	Gene_ID
PhBLH1	PH02Gene06220
PhBLH2	PH02Gene11412
PhBLH3	PH02Gene11465
PhBLH4	PH02Gene11822
PhBLH5	PH02Gene15691
PhBLH6	PH02Gene19423
PhBLH7	PH02Gene23070
PhBLH8	PH02Gene26451
PhBLH9	PH02Gene27351
PhBLH10	PH02Gene32857
PhBLH11	PH02Gene37296
PhBLH12	PH02Gene40057
PhBLH13	PH02Gene22367
PhBLH14	PH02Gene22684
PhBLH15	PH02Gene40432
PhBLH16	PH02Gene43129
PhBLH17	PH02Gene00888
PhBLH18	PH02Gene05329
PhBLH19	PH02Gene15692
PhBLH20	PH02Gene17085
PhBLH21	PH02Gene20655
PhBLH22	PH02Gene22578
PhBLH23	PH02Gene23946
PhBLH24	PH02Gene30743
PhBLH25	PH02Gene31820
PhBLH26	PH02Gene32858
PhBLH27	PH02Gene37932
PhBLH28	PH02Gene42311
PhBLH29	PH02Gene43590
PhBLH30	PH02Gene45309
PhBLH31	PH02Gene46931
PhKNOX1	PH02Gene12434
PhKNOX2	PH02Gene13429
PhKNOX3	PH02Gene20967
PhKNOX4	PH02Gene31875
PhKNOX5	PH02Gene38176
PhKNOX6	PH02Gene45662
PhKNOX7	PH02Gene00891
PhKNOX8	PH02Gene04791
PhKNOX9	PH02Gene23948
PhKNOX10	PH02Gene04354

PhKNOX11	PH02Gene13867
PhKNOX12	PH02Gene25890
PhKNOX13	PH02Gene32186
PhKNOX14	PH02Gene12436
PhKNOX15	PH02Gene25222
PhKNOX16	PH02Gene26384
PhKNOX17	PH02Gene26728
PhKNOX18	PH02Gene26729
PhKNOX19	PH02Gene26730
PhKNOX20	PH02Gene30737
PhKNOX21	PH02Gene36015
PhKNOX22	PH02Gene37175
PhKNOX23	PH02Gene43279
PhKNOX24	PH02Gene46847
