# Supplementary material for: Genome-Wide Identification, Expansion, and Evolution Analysis of Homeobox Gene Family Reveals TALE Genes Important for Secondary Cell Wall Biosynthesis in Moso Bamboo (Phyllostachys edulis)
Source: Int J Mol Sci. 2022 Apr 8;23(8):4112. doi: 10.3390/ijms23084112 (PMC9032839; doi:10.3390/ijms23084112)
Supplement: Supplementary file 1 [file ijms-23-04112-s001.zip › Table S3.pdf]

**Table S3 The collinear relationship of TALE subclass between rice and moso bamboo.**

| Subfamily | Os_Homeobox | Moso_Homeobox | Annotation |
|---|---|---|---|
| **BLH** | Os01g0848400 | PH02Gene11412<br>PH02Gene20655 | *qSH1* |
| **BLH** | Os02g0226600 | PH02Gene05329<br>PH02Gene45309 | ——— |
| **BLH** | Os03g0124000 | PH02Gene00888<br>PH02Gene23946<br>PH02Gene46931 | ——— |
| **BLH** | Os03g0165300 | PH02Gene19423<br>PH02Gene23070<br>PH02Gene40057<br>PH02Gene43590 | ——— |
| **BLH** | Os03g0680700 | PH02Gene15691 | ——— |
| **BLH** | Os03g0732100 | PH02Gene06220<br>PH02Gene40432 | ——— |
| **BLH** | Os05g0455200 | PH02Gene30743<br>PH02Gene37296 | *SH5* |
| **BLH** | Os06g0108900 | PH02Gene17085<br>PH02Gene31820 | ——— |
| **BLH** | Os06g0562300 | PH02Gene42311 | ——— |
| **BLH** | Os10g0534900 | PH02Gene22578 | ——— |
| **BLH** | Os11g0158600 | PH02Gene11465<br>PH02Gene22367<br>PH02Gene22684<br>PH02Gene26451 | ——— |
| **BLH** | Os12g0636200 | PH02Gene11822<br>PH02Gene27351<br>PH02Gene32857 | ——— |
| **KNOX** | Os01g0302500 | PH02Gene25890<br>PH02Gene31875 | *OSH6* |
| **KNOX** | Os03g0123500 | PH02Gene00891<br>PH02Gene23948 | *OsKNAT7* |
| **KNOX** | Os03g0673000 | PH02Gene26384<br>PH02Gene45662 | *OSH10* |
| **KNOX** | Os03g0727000 | PH02Gene12436<br>PH02Gene26728 | *OSH1* |
| **KNOX** | Os05g0129700 | PH02Gene04354<br>PH02Gene25222 | *OSH71* |
| **KNOX** | Os06g0646600 | PH02Gene04791<br>PH02Gene13867 | ——— |

| | | | |
|---|---|---|---|
| | | PH02Gene30737 | |
| | | PH02Gene36015 | |
| | | PH02Gene13429 | |
| **KNOX** | Os07g0129700 | PH02Gene20967 | *OSH15* |
| | | PH02Gene32186 | |
| | | PH02Gene38176 | |
| **KNOX** | Os08g0292900 | PH02Gene37175 | *AtKNAT3* |
| | | PH02Gene46847 | |
| **KNOX** | Os03g0727200 | PH02Gene12434 | ——— |
| | | PH02Gene26729 | |
