# Supplementary material for: Genome-Wide Identification, Expansion, and Evolution Analysis of Homeobox Gene Family Reveals TALE Genes Important for Secondary Cell Wall Biosynthesis in Moso Bamboo (Phyllostachys edulis)
Source: Int J Mol Sci. 2022 Apr 8;23(8):4112. doi: 10.3390/ijms23084112 (PMC9032839; doi:10.3390/ijms23084112)
Supplement: Supplementary file 1 [file ijms-23-04112-s001.zip › Table S5.pdf]

Table S5 Primers used to perform qRT-PCR.

Gene_sym	Primer sequences (5'-3')
PhBLH1	F--GCTCAGTCACCGTCATCCG<br>R--CCATCACCGAGTCGAAGAAGT
PhBLH2	F--TGGTTCCCAGAGTTTCCC<br>R--CGCCGACTTGCTGATGT
PhBLH3	F--TCCTCAGCCTGTCATCCCG<br>R--CCCTTGCTAACGCTCACCAC
PhBLH4	F--TCCTGGCACCTTCATTAC<br>R--CCTCATCGAGCAGTTCTT
PhBLH5	F--CTTACATCATACCGAGAACA<br>R--AACACCGATAGGAGCAG
PhBLH6	F--AATCCTCAACGGTGGGTCTT<br>R--TGTTCGTGGTCGGCTCCT
PhBLH7	F--AGCATTTCCTCCAACCGTATC<br>R--CGATCATCGGCTTCCACAG
PhBLH8	F--CGTGCTGAGCCTGTCGTTC<br>R--TCGCCTCCTCTACACCCTTG
PhBLH9	F--GATGATGAAGGACCAACG<br>R--GTACCTCCCAAGCTCTGC
PhBLH10	F--CAAACTCCTCCGACAACG<br>R--ACCAAGACTCACCATCCC
PhBLH11	F--AATGCAAGGGTTAGAGTATGG<br>R--TCTGACGAATGCTGGGTTT
PhBLH12	F--TCGTTCCACCACTACCCTCC<br>R--CGTTGTCCGTCGGTCCTT
PhBLH13	F--GCACTTCCTACACCCATACCC<br>R--TCCACCATCGGCTTCCA
PhBLH14	F--GAAGACTCGGAAGGTGGGT<br>R--CCTGCATCTGGTGGTGGT
PhBLH15	F--GGCATGATGGAGCAGGAGG<br>R--CGGACACGGGCGTTGAT
PhBLH16	F--GCCGACTTGTAGCAGTGGAGG<br>R--TTGGATACGGGTGAAGGAAATG
PhKNOX1	F--CAGGGAGCAGCAGCAAGA<br>R--CAGTAGGACCACCGGATGTAG
PhKNOX2	F--TGGAGGAGGGAGCGGTAGT<br>R--GCCATGCTGCATCGACAA
PhKNOX3	F--TACTTGGGCAACCTCCG<br>R--GCGATCTTATCCGTCTCA
PhKNOX4	F--TGCGGTACAAGGAGGAGC<br>R--CATTTCATCGGAGTGGGTG
PhKNOX5	F--CCGCCTGAGATTGACCC

	R--TCCCACCAGTGGAGTAGCT
PhKNOX6	F--TTAGGACTATGGAGGCG
	R--GGAGGTGGTTCTTCAGC
PhKNOX7	F--AGCCGCAGATAATGGATG
	R--CAATTCTCGACTTGAAACCC
PhKNOX8	F--CGTGTTGCTCCTTTGCT
	R--TCGCTCCAGTGCCTTCA
PhKNOX9	F--CCCCACGGAAGACGATA
	R--CGCTTTGATTTAAGGGTTGA
PhKNOX10	F--CCTTCGTTCCGAGTTCTT
	R--GGCGAGCCTCACCTTAT
PhKNOX11	F--GTATTGCTCCTTTGTTCG
	R--ATAGTTGCTCCAGTGCC
PhKNOX12	F--ACTTCGGTCCGAGTTTCT
	R--CGAGCCTCACCTTATCTTC
PhKNOX13	F--GAGCCACCTGAGATTGACC
	R--TTTGGGAGCTTCCCTTTC
PhXOAT3-1	F--ACTGGGATTGGCATCTTGACG
	R--GGAGTTGACGAGGCAGACGAA
PhXXT	F--GAGTATGGAGCGAGCATTTAA
	R--AGGCTTGGTGGTCTGATTT
PhIRX10-2	F--CCCCAGCAAATACAACA
	R--GCAAGGGATGACCAGAA
PhIRX15-2	F--GGCTCGGGTTTGGCCTCCTA
	R--TGTCCGTCATGCGGTGGGTG
PhKOBITO1	F--GTCCTACTTCGCCGTCTC
	R--GATGTGCTTCCCGTGATA
PhIRX10-3	F--GGAAGGCTCCATCACCA
	R--TGCCGTATCATAGAATAAACC
PhIRX10-4	F--GAGGAGGTGGGTCTTGG
	R--CAGCAGCTTCTTGTTGTATTT
PhCESA-1	F--CGTCAAGGAGCGTAGGG
	R--AGGGTGGTCACGAGGGT
PhCESA-2	F--ATCGCTCGCAGAAACGG
	R--CCCTACGCTCCTTGACG
PhNAC43-4	F--CCATCTACAACACCGTCAAGC
	R--ACACCACCCAGCCATCCT
PhXOAT3-2	F--CACGCCCAAATCGACCAC
	R--CTGATGCCGCCTTCCCT
PhCESA-6	F--GGGTGAGCATCGAAGATTGGT
	R--GAGGTGACGGTGAAGTTGGTGT
PhCESA-7	F--GTGAGCGGTGAGTTTCCA
	R--TTCTTCTCGTCCCACTTTG
PhCASD1-2	F--CATCATAGCATCGGCACT

	R--TCTGAGGCAGCGAAATAG
PhSEY1	F--TGGAAGGCACTGATGGA<br>R--TGCGAGGACTGAACAACC
PhGXM-2	F--ACGCCGACGACCTCATCTC<br>R--ATTGCGCCCATCCTCCC
PhCASD1-1	F--ATGCCCTATGACAACCC<br>R--CACCAGGAATTTCCCAC
PhIRX9-4	F--GCCTGTGGCGACGATGT<br>R--CAAGTCAGCCTGCGTGTTAT
PhIRX9-5	F--CTCTTCCACCATATTCGTCA<br>R—CAGCCTCTTGTTCTTCTCG
PhIRX6-2	F--GGCATGTTCTACGGGCTCA<br>R--GCGGCATCTTGCACTCG
PhIRX6-3	F--CGCAAAGACGCCAACACC<br>R--CAGCAAGAACGCCGAAGC
PhCESA-4	F---AACAACCTGGCGAGTATCTGG<br>R---CCTTGGAGGTGACGGTGAA
