# Supplementary figures and images for: Genome-Wide Identification, Expansion, and Evolution Analysis of Homeobox Gene Family Reveals TALE Genes Important for Secondary Cell Wall Biosynthesis in Moso Bamboo (Phyllostachys edulis)
Source: Int J Mol Sci. 2022 Apr 8;23(8):4112. doi: 10.3390/ijms23084112 (PMC9032839; doi:10.3390/ijms23084112)

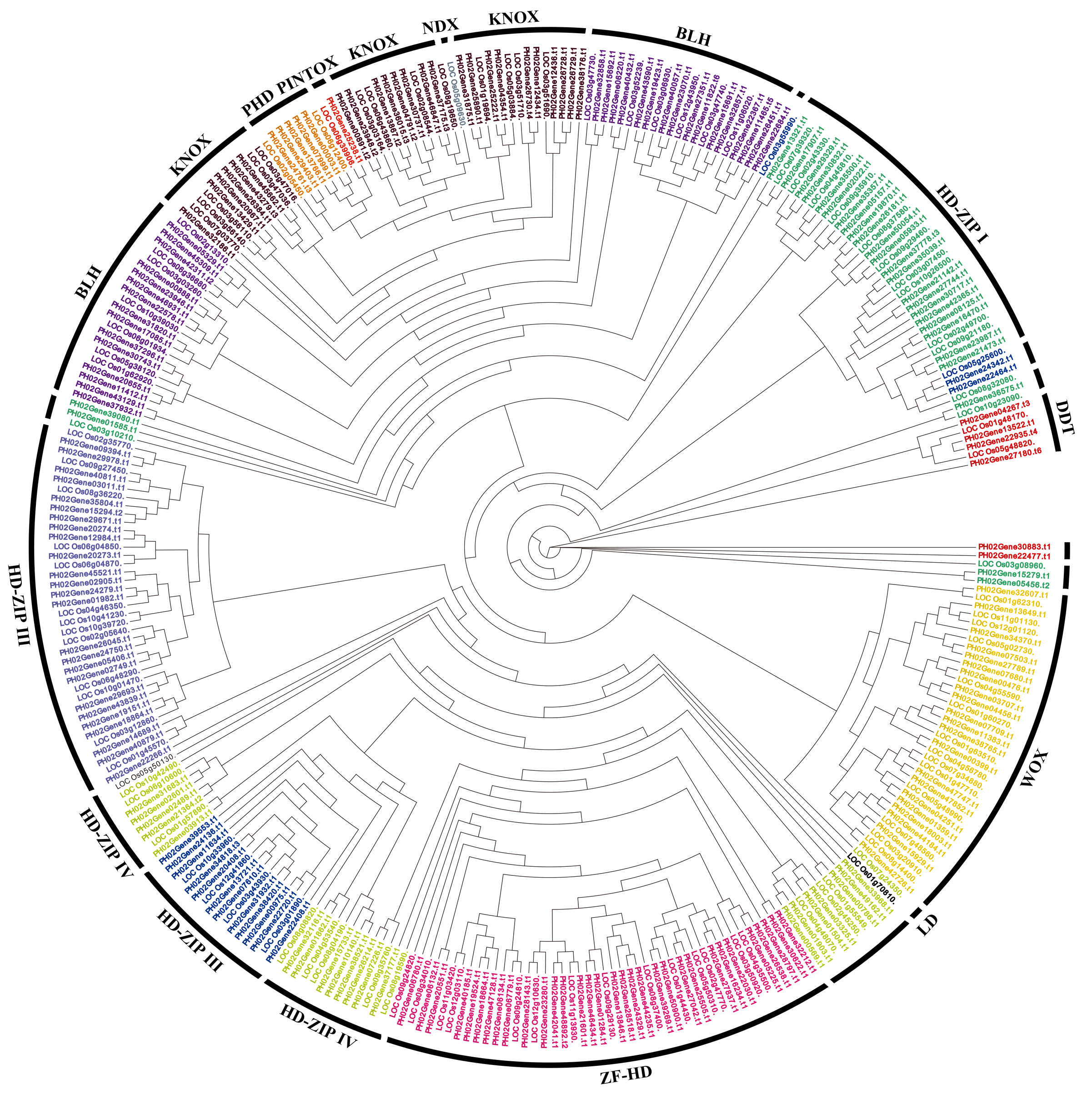

Supplement: Supplementary file 1 [file ijms-23-04112-s001.zip › Figure S1.tif]

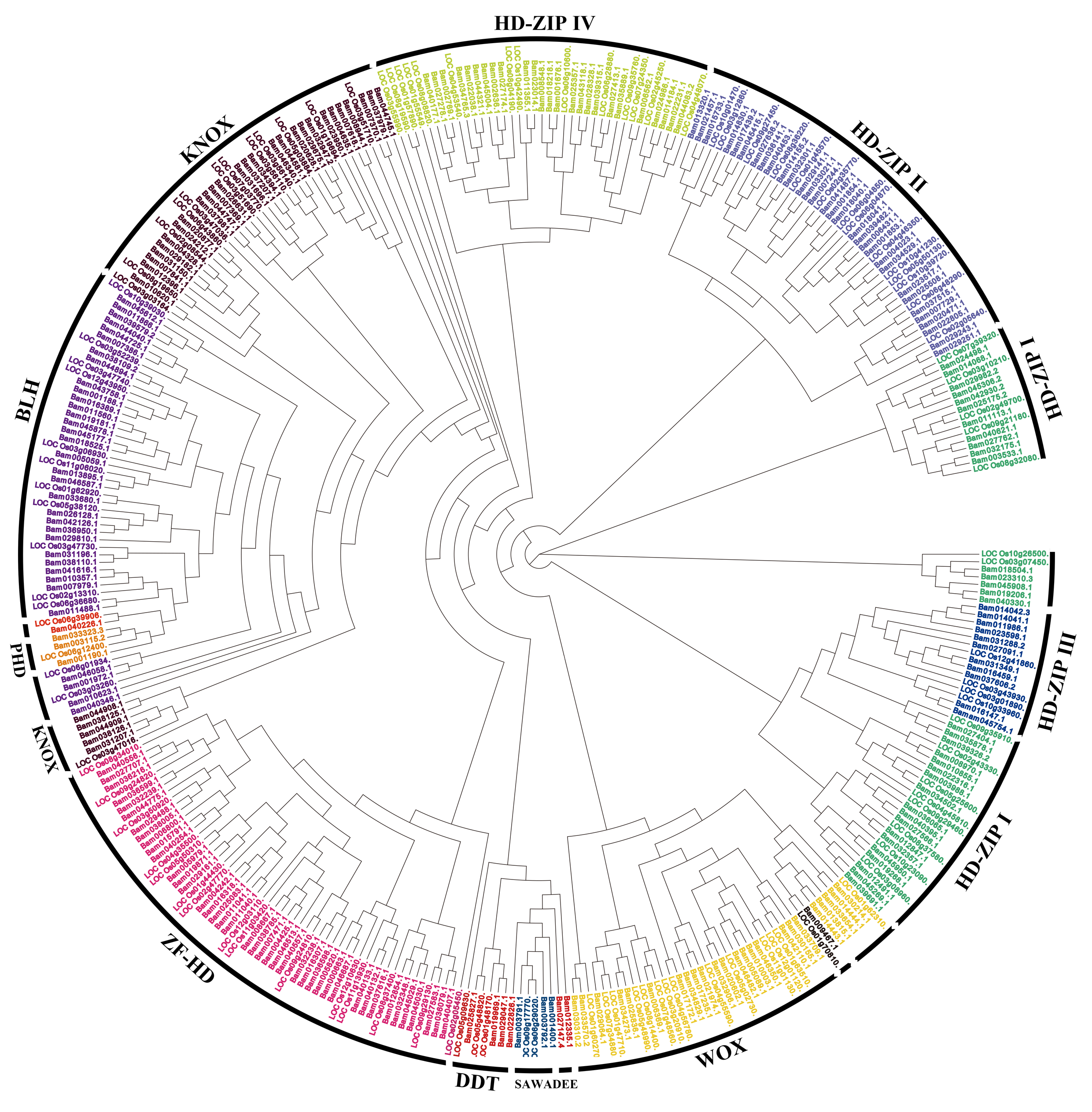

Supplement: Supplementary file 1 [file ijms-23-04112-s001.zip › Figure S2.tif]

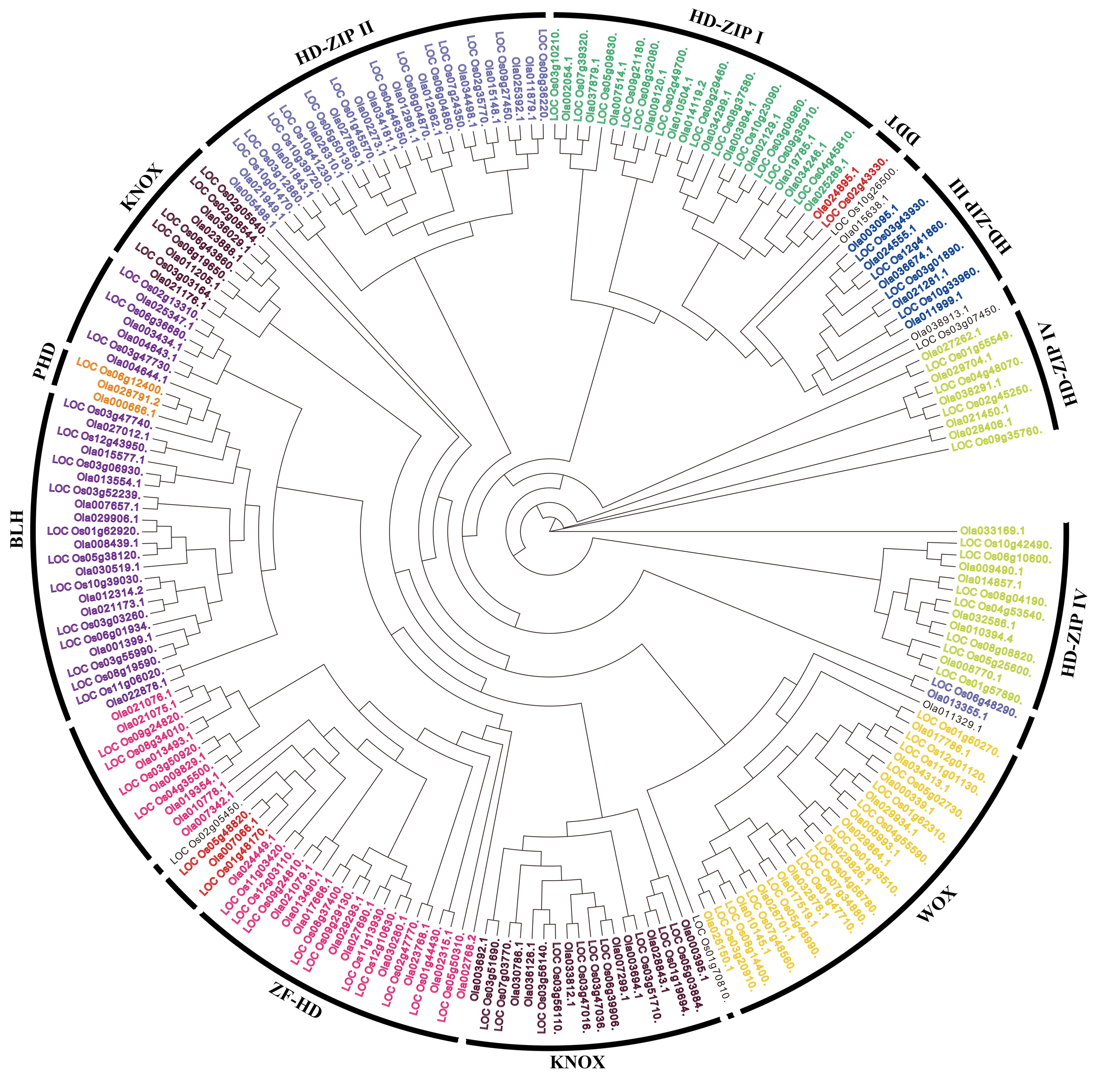

Supplement: Supplementary file 1 [file ijms-23-04112-s001.zip › Figure S3.tif]

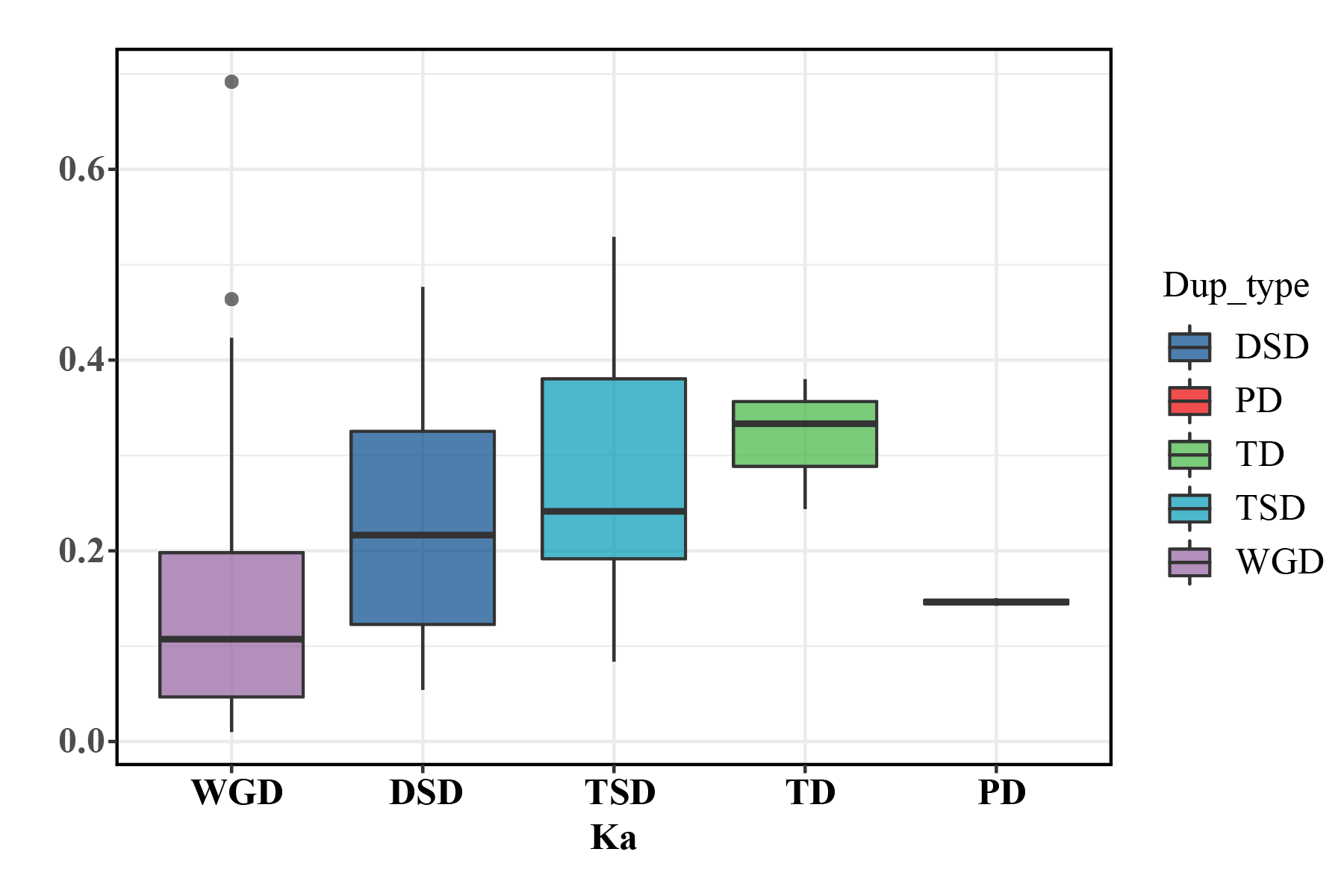

Supplement: Supplementary file 1 [file ijms-23-04112-s001.zip › Figure S4.tif]

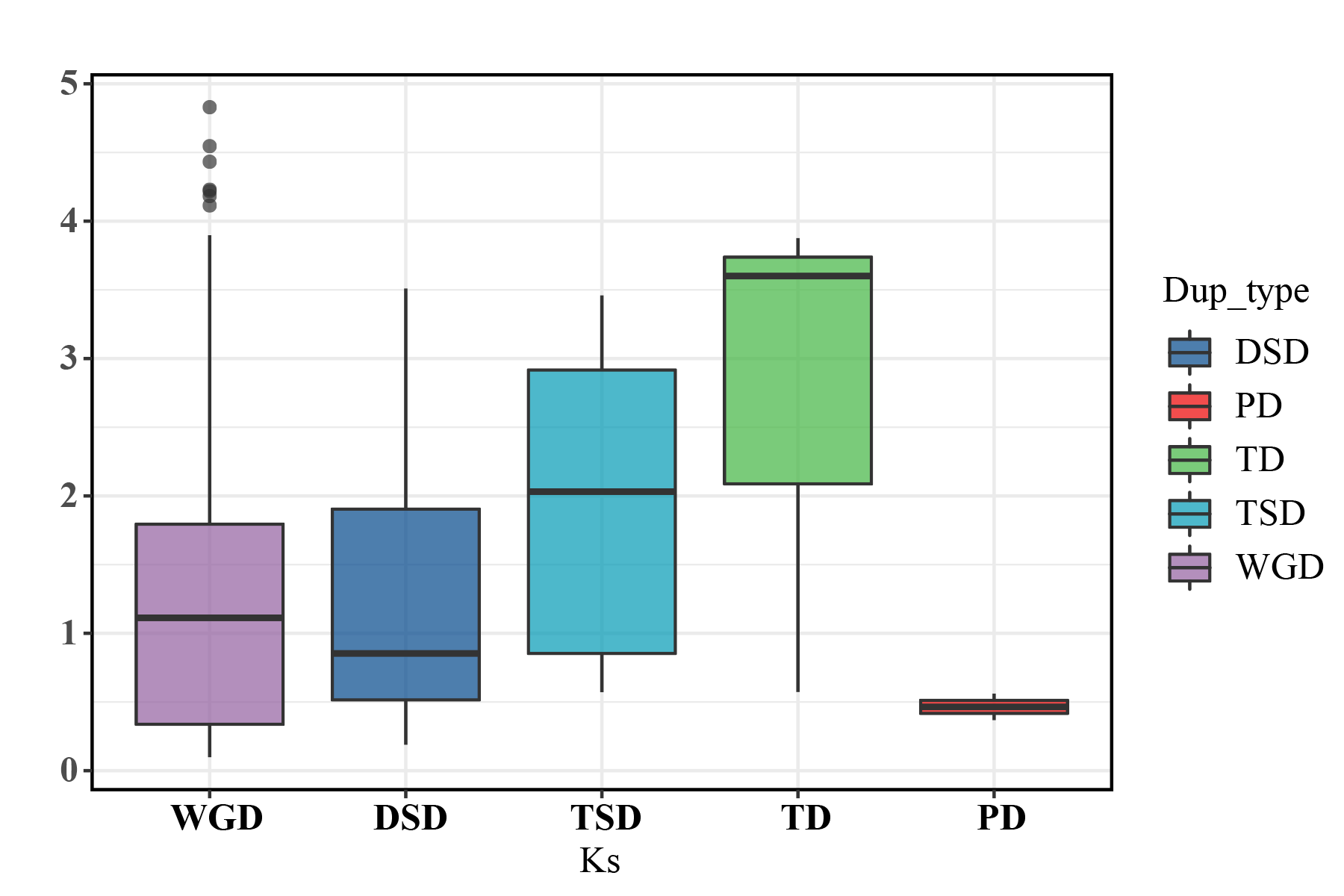

Supplement: Supplementary file 1 [file ijms-23-04112-s001.zip › Figure S5.tif]

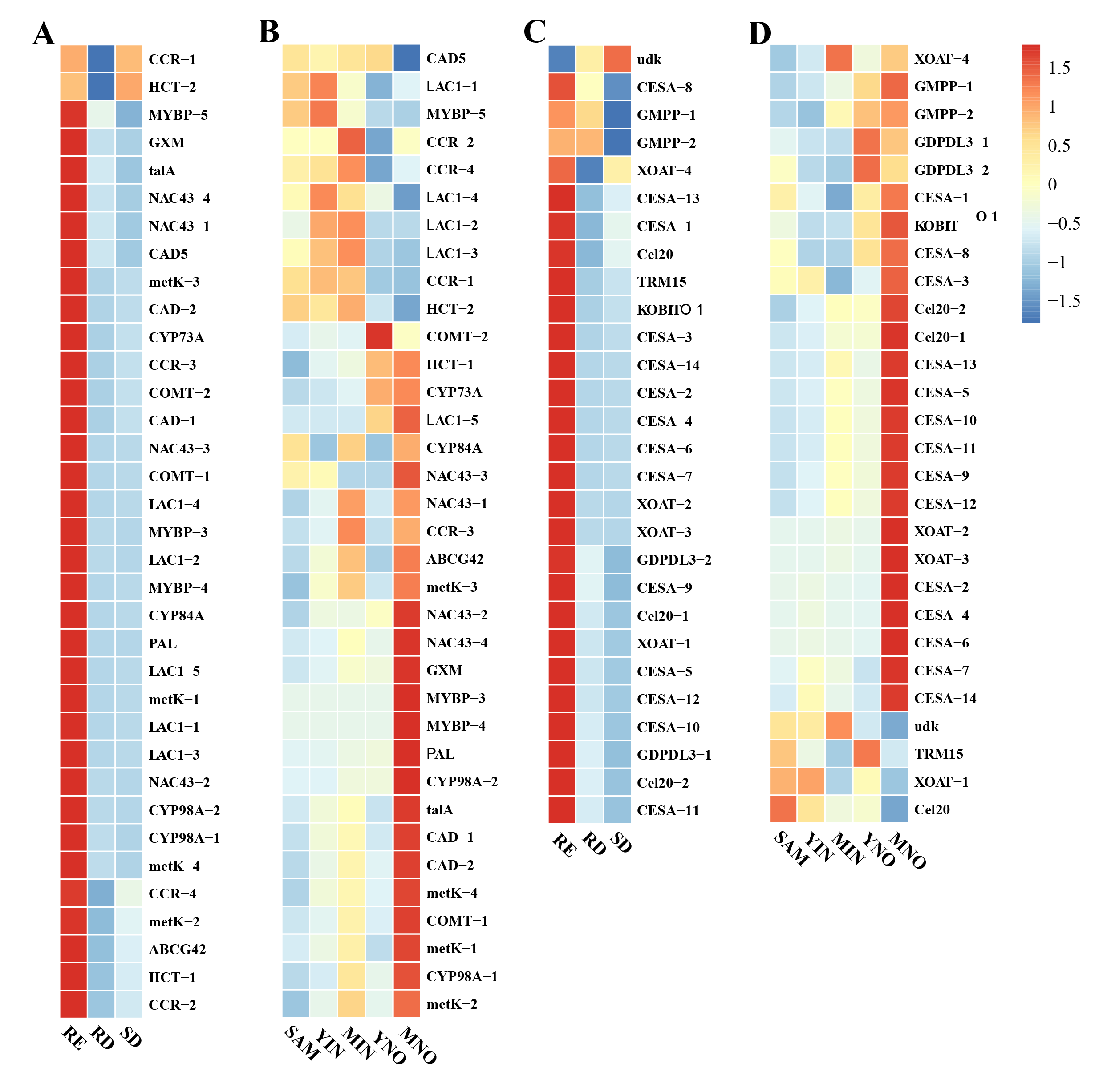

Supplement: Supplementary file 1 [file ijms-23-04112-s001.zip › Figure S6.tif]

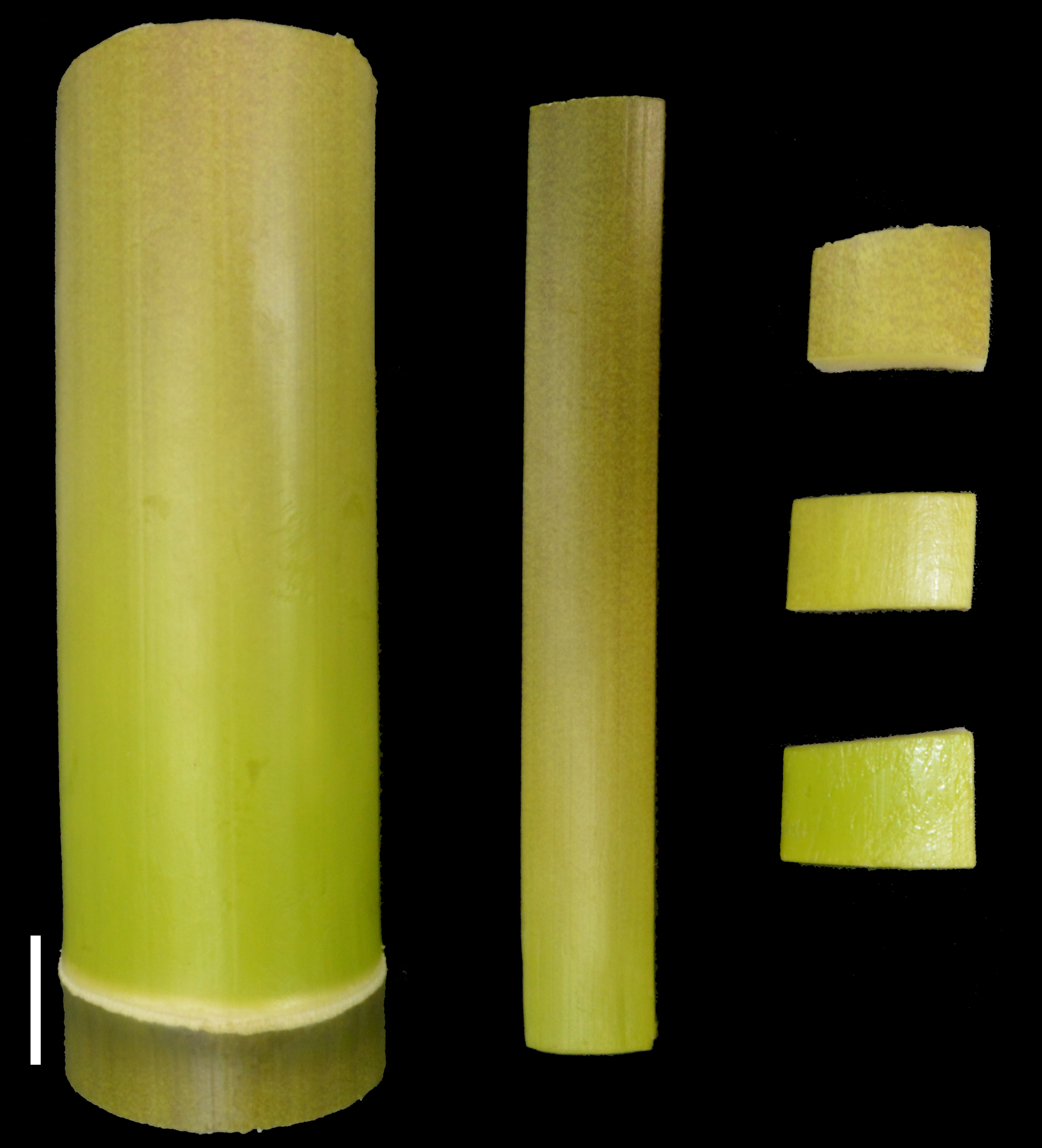

Supplement: Supplementary file 1 [file ijms-23-04112-s001.zip › Figure S7.tif]
